# Characterization of Two Immunodominant Antigenic Peptides in NSP2 of PRRSV-2 and Generation of a Marker PRRSV Strain Based on the Peptides

**DOI:** 10.3389/fvets.2022.902822

**Published:** 2022-05-30

**Authors:** Dong-Yan Li, Xing-Yang Cui, Xin-Yi Huang, Yue Hu, Xiao-Xiao Tian, Tao Wang, Yong-Bo Yang, Qian Wang, Zhi-Jun Tian, Xue-Hui Cai, Tong-Qing An

**Affiliations:** State Key Laboratory of Veterinary Biotechnology, Harbin Veterinary Research Institute, Chinese Academy of Agricultural Sciences, Harbin, China

**Keywords:** porcine reproductive and respiratory syndrome virus, differentiating infected from vaccinated animals vaccine, non-structural protein 2, peptides, enzyme-linked immunosorbent assay

## Abstract

Porcine reproductive and respiratory syndrome (PRRS) is a widespread disease with great economic importance in the pig industry. Although vaccines against the PRRS virus (PRRSV) have been employed for more than 20 years, differentiating infected from vaccinated animals remains challenging. In this study, all 907 non-structural protein 2 (NSP2) full-length sequences of PRRSV-2 available from GenBank were aligned. Two peptides, at positions 562–627 (m1B) and 749–813 (m2B) of NSP2, were selected, and their potential for use in differential diagnosis was assessed. Both m1B and m2B were recognized by PRRSV-positive pig serum in peptide-coated enzyme-linked immunosorbent assays. Further epitope identification yielded five overlapping short peptides for the immunodominant regions of m1B and m2B. Using the infectious clone of PRRSV HuN4-F112 as a template, the deletion mutants, rHuN4-F112-m1B, rHuN4-F112-m2B, and rHuN4-F112-C5-m1B-m2B, were generated and successfully rescued in Marc-145 cells. Growth kinetics revealed that the deletion of m1B and m2B did not significantly affect virus replication. Hence, m1B and m2B show potential as molecular markers for developing a PRRSV vaccine.

## Highlights

- NSP2-specific peptides m1B and m2B react with PRRSV-positive pig serum.
- Deletion of m1B and m2B has no significant effect on virus replication.
- Peptides m1B and m2B can serve as markers in a diagnostic PRRSV DIVA vaccine.

## Introduction

Porcine reproductive and respiratory syndrome (PRRS) is an important viral disease that causes reproductive disorders in sows and respiratory disorders in pigs of all ages (1). Since its discovery in the United States in 1987 and Europe in 1990, the disease has led to huge economic losses in the pig industry (2–4). Genomic sequencing has identified two PRRS virus (PRRSV) genotypes, PRRSV-1 (European type) and PRRSV-2 (North-American type) (5–7). PRRSV is a positive single-stranded RNA virus containing a 5′-untranslated region and at least 10 open reading frames (ORFs), including ORF1a, ORF1b, ORF2a, ORF2b, ORF3, ORF4, ORF5, ORF6, ORF7, and ORF5a (8). The polyproteins pp1a and pp1ab, encoded by ORF1a and ORF1b, respectively, undergo hydrolysis to produce 14 non-structural proteins (NSPs), NSP1α, NSP1β, NSP2–NSP6, NSP7α, NSP7β, NSP8–NSP12, and NSP2TF (9–11). Although NSP2 is the largest replicase of PRRSV, its gene exhibits significant diversity among different PRRSV strains (12). Mutations, insertions, and deletions are common in the middle region of NSP2 (13, 14).

The first PRRSV was isolated in mainland China in 1996 (15), after which the disease spread to all main pig-rearing provinces in the country. In 2006, a more serious and highly pathogenic PRRSV (HP-PRRSV) variant, characterized by a 30-amino acid deletion in NSP2, triggered an outbreak with high morbidity and mortality among pigs of all ages in some Asian countries (16). Subsequent experiments showed that the 30-amino acid deletion in NSP2 was not related to the pathogenicity of HP-PRRSV (17). The emergence of NADC30-like PRRSV in 2013 was the cause of another epidemic in China (18, 19), which has resulted in increased abortions among pregnant sows. NADC34-like PRRSV strains were identified in China in 2017, and new studies have recently reported that NADC34-like PRRSV strains exhibit complex recombination patterns with NADC30-like and HP-PRRSV (20, 21).

Vaccination is a key strategy for controlling the spread of PRRSV. Approximately 10 vaccines against PRRSV have been licensed, including killed vaccines and modified live vaccines (MLVs). In general, the latter provide better immunological protection than the former but cannot readily distinguish between infected and vaccinated animals. To identify potential positions in NSP2 at which to introduce molecular markers, NSP2 sequences were systematically compared, and two universal regions were selected for antigenic analysis. Importantly, the two regions were deleted without affecting the growth of the PRRSV MLV HuN4-F112 strain *in vitro*, suggesting that these regions were suitable for developing vaccines capable of differentiating infected from vaccinated animals (DIVA).

## Materials and Methods

### Cells, Virus, and Antibodies

Marc-145 cells were grown in Dulbecco's modified Eagle's medium (Invitrogen, Carlsbad, CA, United States) supplemented with 10% fetal bovine serum (Gibco, Invitrogen, Gaithersburg, MD, United States) at 37°C with 5% CO_2_. The modified live vaccine HuN4-F112 strains, the infectious clones of HuN4-F112 and HuN4-F112-C5, were generated in our previous study (18, 22). PRRS-positive serum collected from pigs experimentally infected with the HeB108 strain (NADC30-like PRRSV) or HLJ13 strain (NADC34-like PRRSV), PRRS-positive serum collected from pigs vaccinated with HuN4-F112 (HP-PRRSV) or CH-1R (classical PRRSV), and PRRS-negative pig serum were maintained in our laboratory. Positive pig serum against classical swine fever virus, porcine epidemic diarrhea virus, transmissible gastroenteritis virus, porcine circovirus type 2, pseudorabies virus, and porcine parvovirus, as well as a monoclonal antibody (3F7) against the M protein of PRRSV, were obtained from our laboratory (23).

### Dataset and Alignment of NSP2 From PRRSV-2

In our previous study, two amino acid residues at positions 585–586 in the NSP2 of PRRSV HeB108 were deleted during viral passage in Marc-145 cells (unpublished data), indicating that these or nearby residues can be used as markers for next-generation DIVA vaccines. To identify the deletions in circulating PRRSV and MLV strains, all 907 NSP2 full-length sequences of PRRSV-2 available from GenBank in 2020 were downloaded, followed by amino acid sequence alignment and insertion/deletion (indel) analysis using DNASTAR software v7.1.0 (DNASTAR, Madison, WI, United States).

### Immunoreactivity of m1B and m2B Peptides

Indirect enzyme-linked immunosorbent assay (ELISA) was used to evaluate the immunoreactivity of m1B and m2B peptides. All peptides employed in the study were synthesized by GL Biochem, Ltd. (Shanghai, China). ELISA plates were coated with m1B or m2B peptides alone or in combination at a dose of 10 μg/well *via* incubation in carbonate-bicarbonate buffer [15 mM Na_2_CO_3_, 35 mM NaHCO_3_ (pH 9.6)] at 4°C overnight. The plates were then blocked with 5% non-fat dry milk in phosphate-buffered saline (PBS) containing 0.05% Tween-20 (PBST) for 1 h at 37°C. After washing three times with PBST, 100 μl of PRRSV-positive pig serum (1:40 dilution) was added to the wells. The plates were incubated, followed by incubation at 37°C for 1 h, washed again, and then incubated with horseradish peroxidase-conjugated rabbit anti-pig IgG (1:40,000 dilution; Sigma-Aldrich, St. Louis, MO, United States) in PBST at 37°C for 1 h. Finally, the plates were washed and incubated with 100 μl/well of TMB (Solarbio, Beijing, China) for 15 min. The reaction was stopped with 2M H_2_SO_4_ (100 μl/well), and the results were read at 450 nm using a Microplate Absorbance Reader (Bio-Rad, Hercules, CA, United States). Sixty-five serum samples, with varying OD values, were probed in the ELISA. The samples with a P/N ratio >2 were considered to be positive.

### Assessment of Specificity

The specificity of the 1B2B-ELISA indirect ELISA was examined using the antisera of the six porcine viruses mentioned previously herein to assess the degree of assay cross-reactivity.

### Immunoreactivity of m1B and m2B Truncated Peptides

To identify the immunodominant antigen regions in the m1B and m2B peptides, seven overlapping peptides (m1B1–m1B7) spanning the m1B region were designed. Each peptide was 16 residues long, and the overlapping region between two adjacent peptides spanned eight residues (Table 1). Similarly, seven overlapping peptides (m2B1–m2B7) were designed and synthesized to probe the m2B region. The resulting m1B1–m1B7 and m2B1–m2B7 peptides were used as coating antigens in ELISA, and their reactivity with PRRSV-positive serum was detected as described previously herein.

**Table 1:** Amino acid sequences of short-peptides in this study.

**Peptide name**	**Sequence**	**Positions in NSP2**
m1B1	TTTLTHQDEPLDLPAS	562–577
m1B2	PLDLPASSQTEYEAFP	570–585
m1B3	SQTEYEAFPLAPSQNM	578–593
m1B4	LAPSQNMGILEAGGQE	586–601
m1B5	GILEAGGQEVEEVLSEI	594–609
m1B6	VEEVLSEISDILNDTN	602–617
m1B7	SDILNDTNPAPVSSSSS	611–627
m2B1	IGSVATEDVPRILGKIG	749–764
m2B2	PRILGKIGDTDELLDR	757–772
m2B3	DTDELLDRGPSAPSKG	765–780
m2B4	GPSAPSKGEPVCDQPA	773–788
m2B5	EPVCDQPAKDPRMSPR	781–796
m2B6	KDPRMSPRESDESMIA	789–804
m2B7	ESDESMIAPPADTGGVG	797–813

### Generation of PRRSV m1B or m2B Deletion Mutants

Plasmids harboring m1B or m2B deletion mutants were constructed using HuN4-F112 as a template and specifically designed primers (Table 2); a plasmid harboring the m1B and m2B double-deletion mutant was constructed using HuN4-F112-C5 as a template and specifically designed primers. The native m1B and m2B genes in the HuN4-F112 infectious clone were replaced with their deletion variants *via* overlap PCR amplification using ultra-fidelity DNA polymerase (TaKaRa, Shiga, Japan) as described previously (24), followed by digestion with restriction enzymes (*Fse* I and *Nhe* I, TaKaRa, Dalian, China) and ligation. The deletions were confirmed by DNA sequencing. The plasmids were transfected into Marc-145 cells using Xtreme GENE-HP DNA transfection reagent (Roche Applied Science, Basel, Switzerland) as described previously (25).

**Table 2:** Primers used in this study.

**Primer name**	**Sequence**
H-B-FseI-F	GAGTGGGCCGGCCCCAGT
I-B-NheI-R	GCCGCTGGCGGCTAGCAG
H-B-m1B-F	GTGGCGGCCGCTCAAGTGTTAAGATCA
I-B-m2B-R	CGGCAAATCAGTGAATGAAATGGTGAGGTCACTCTCT
H-B-m2B-F	GAGTGACCTCACCATTTCATTCACTGATTTGCCGTCT
H-1B-2B-F	CAGAGAGTGACCTCACCATTTCATTCACTGATTTGCCG
H-1B-2B-R	CGGCAAATCAGTGAATGAAATGGTGAGGTCACTCTCTG
B-900-F	AGTGAGCCCGTACTTATG
B-900-R	ACCCCCATTCGCACACG

At 5 days post-transfection, the cells were assayed by indirect immunofluorescence (26). Marc-145 cells were incubated with a monoclonal antibody against the M protein of PRRSV and stained with fluorescein isothiocyanate-labeled anti-mouse IgG (Sigma-Aldrich, St. Louis, MO, United States). The cells were observed with an inverted fluorescence microscope (Olympus, Tokyo, Japan).

The supernatant was recovered and passaged twice in Marc-145 cells and identified by reverse transcription PCR. Viral RNA from rHuN4-F112, rHuN4-F112-m1B, rHuN4-F112-m2B, rHuN4-F112-C5, and rHuN4-F112-C5-m1B-m2B were extracted using a QIAamp Viral RNA Mini Kit (Qiagen, Hilden, Germany). Reverse transcription reactions were performed at 25°C for 10 min and 42°C for 1 h using the M-MLV Reverse Transcription Polymerase System (TaKaRa, Dalian, China) and then amplified with B-900-F/B-900-R primers (Table 2), and the PCR products were sent for DNA sequencing (Comate Bioscience Co., Ltd. Bioscience Co., Ltd., Changchun, China.). Viral rescue was quantified in Marc-145 cells.

### Replication Kinetics of Deletion-Marker Viruses in Marc-145 Cells

To monitor the effect of deletion on viral replication, Marc-145 cells were grown to 80% confluence in a six-well plate and then infected with the third-passage viruses (rHuN4-F112-C5 and rHuN4-F112-C5-m1B-m2B) at a multiplicity of infection of 0.01. The supernatant was harvested at 0, 24, 48, 72, and 96 h post-inoculation. After RNA extraction and reverse transcription, the viral copy numbers at different time points were determined by quantitative reverse transcription PCR (RT-qPCR) as described previously (27). All samples were tested three times, and the replication kinetic curve was drawn using GraphPad software (GraphPad, Inc., La Jolla, CA, United States). Measured values were expressed as the mean ± standard deviation. Significance was assessed using a Student's *t*-test at *P* < 0.05.

## Results

### Indel Polymorphic Analysis of NSP2

**Figure 1:**
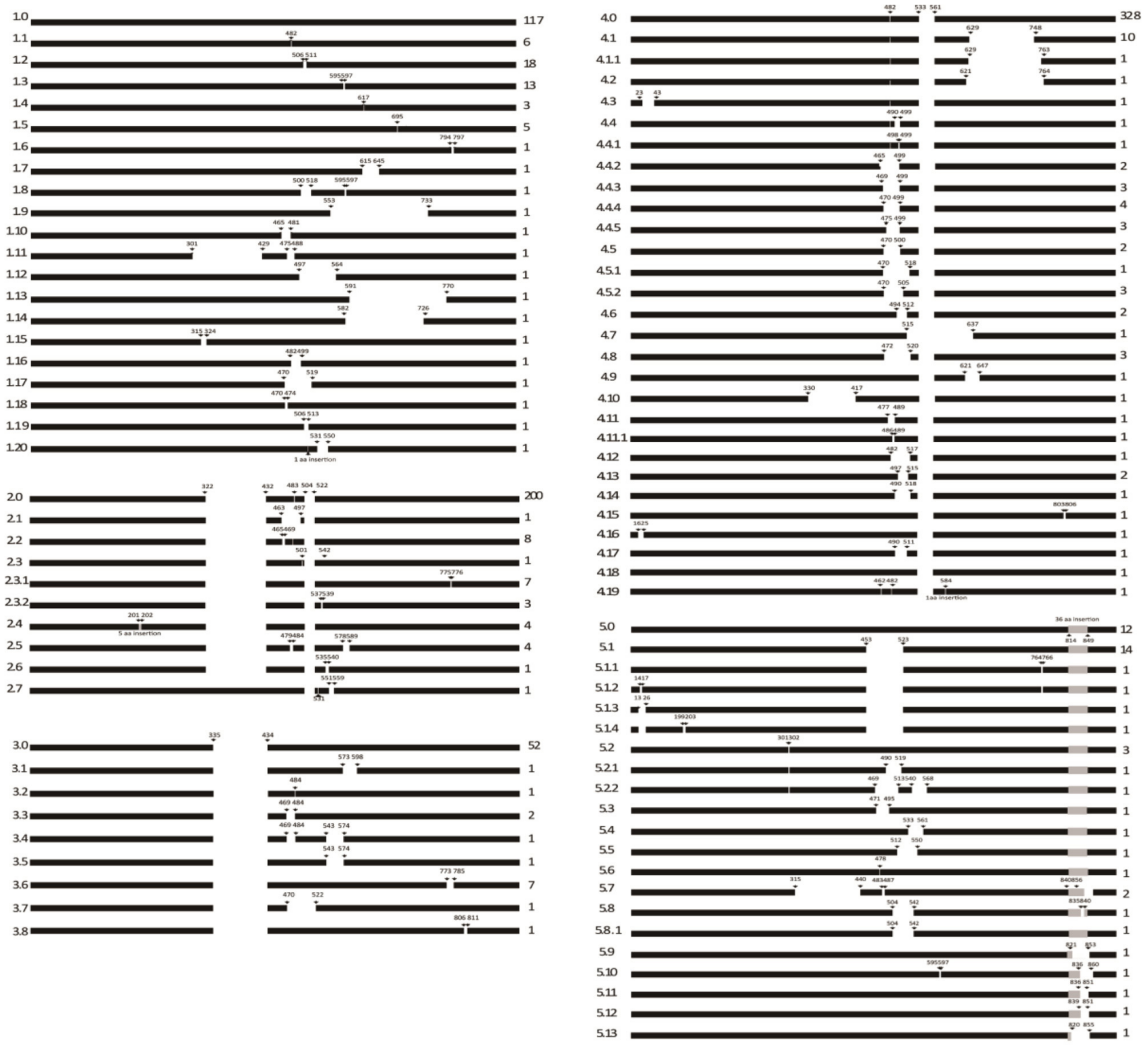
Systematic indel patterns of NSP2 based on 907 PRRSV-2 sequences. The indel patterns of NSP2 are divided into five main categories. The arrows and numbers indicate the indel locations in NSP2 using the position in the VR-2332 strain as a reference. Values on the right represent the numbers of strains.

**Figure 2:**
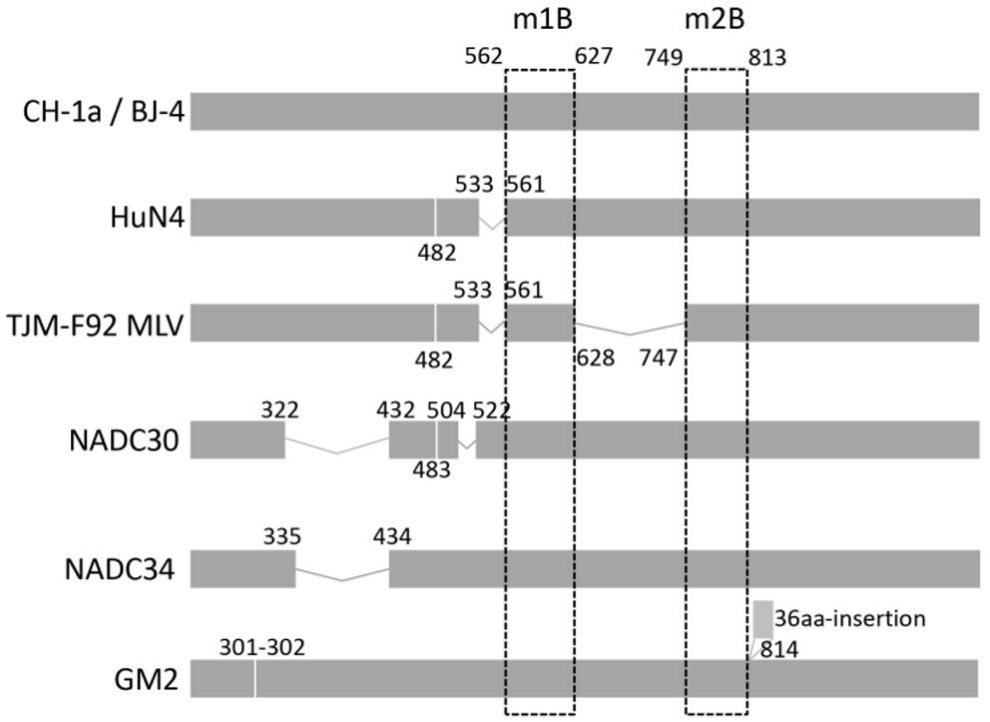
Insertions and deletions in NSP2 of circulating PRRSV-2 or the MLV strain. VR-2332, the prototype of PRRSV-2, was used as a representative strain. Strains are listed on the left. The positions labeled in the figure are preferred to the corresponding position in VR-2332. Two regions (named m1B and m2B) at 562–627 and 749–813 are universal in PRRSV-2. On the left is the name of the representative strain of each indel type.

To systematically analyze the indel polymorphism of NSP2, all 907 NSP2 full-length sequences of PRRSV-2 deposited in GenBank between 1991 and 2020 were aligned. Using VR-2332 as a reference strain, extensive indels were found in NSP2 (Figure 1). They were divided into five main patterns as follows: classic PRRSV (no indels), NADC30 (deletion at positions 322–432, 483, and 504–522), NADC34 (deletion at position 335–434), HP-PRRSV (deletion at positions 482 and 533–561), and SP (36-amino acid insertion at position of 814). All five main indel patterns were detected in PRRSVs isolated in China. During indel analysis, deletions at position 585–586 were found to be somewhat unique. To investigate whether the deletion could be used to design a DIVA vaccine, the antigenic potential of the m1B peptide, for which the start-stop sequence spanned positions 562–627 (Figure 2) and thus covered residues 585–586, was assessed. The same approach was applied with peptide m2B, for which the start-stop sequence spanned positions 749–813 in PRRSV-2.

### Peptides m1B and m2B React With PRRSV-Positive Serum

**Figure 3:**
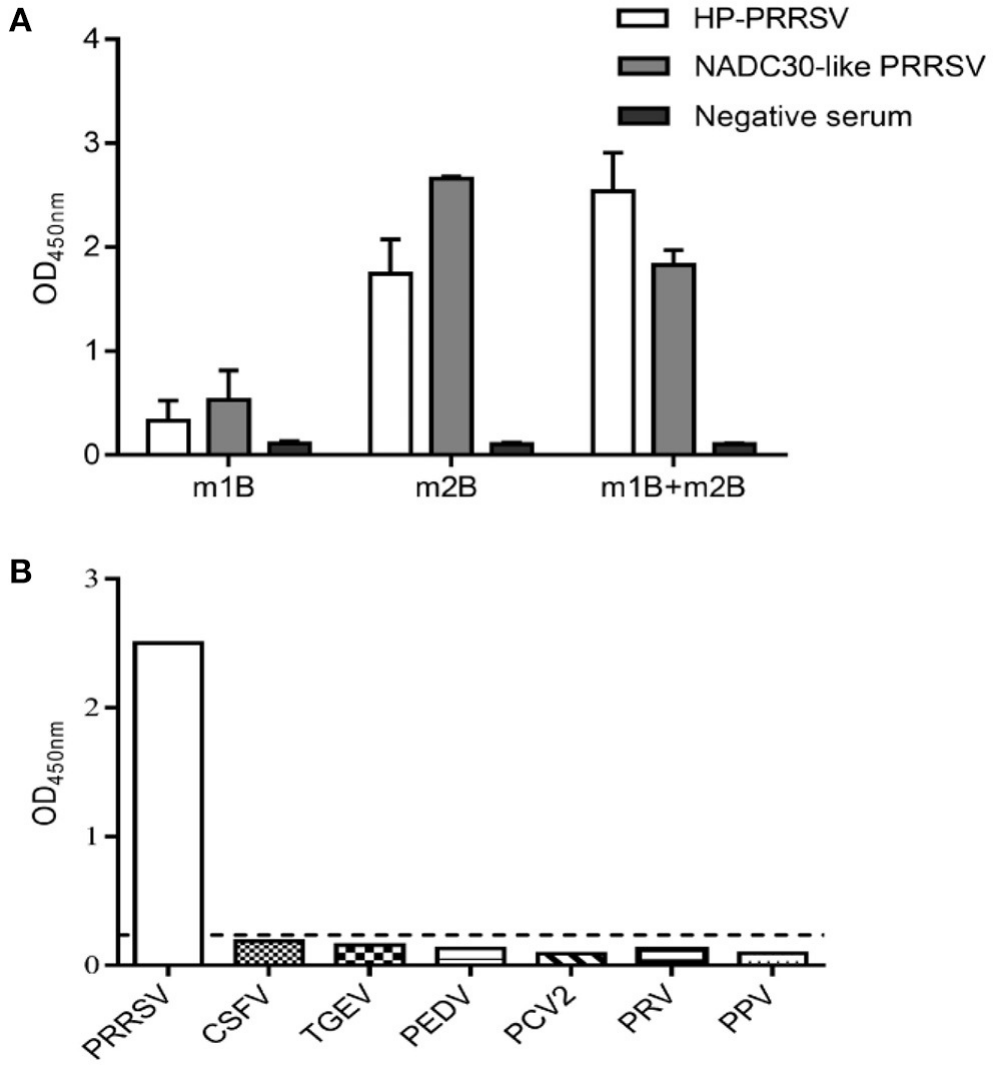
Antigenicity analysis and cross-reactivity assessment of m1B and m2B. **(A)** Indirect ELISA of m1B or m2B peptides. ELISA plates were coated with synthesized peptides, whereas pig serum positive for HP-PRRSV or NADC30-like PRRSV was used as the primary antibody. **(B)** ELISA plates were coated with synthesized peptides, whereas positive serum for PRRSV, CSFV, PEDV, PRV, PPV, and PCV2 was used as the primary antibody. The black dotted line represents the cutoff value of 1B2B-ELISA. HP-PRRSV, highly pathogenic PRRSV; CSFV, classical swine fever virus; PEDV, porcine epidemic diarrhea virus; PRV, pseudorabies virus; PPV, porcine parvovirus; and PCV2, porcine circovirus type 2.

To inspect the antigenicity of m1B and m2B, the corresponding peptides were chemically synthesized and employed to coat an ELISA plate. Pig serum-positive PRRSV was used as the primary antibody. Using indirect ELISA, both m1B and m2B were shown to react with both sera, with m2B reacting more strongly (Figure 3A).

### No Cross-Reaction With Other Antisera

The specificity of 1B2B-ELISA indirect ELISA was further examined using positive sera containing antibodies for other swine viral pathogens. No cross-reactions were found, as all values were below the cutoff point (Figure 3B).

### Identification of Immunodominant Antigen Regions in m1B and m2B Peptides

**Figure 4:**
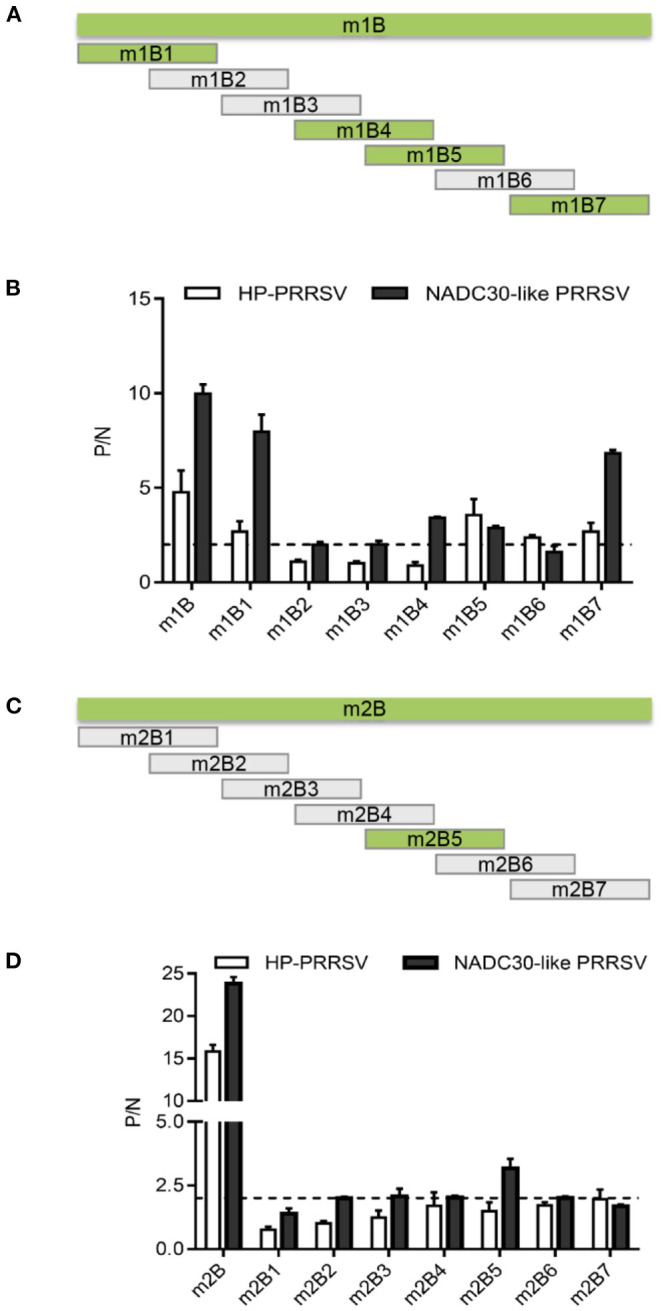
Immunoreactivity of m1B and m2B truncated peptides. **(A)** Schematic diagram of overlapping m1B1–m1B7 short peptides used for epitope mapping. The green box represents peptides that reacted strongly with PRRSV-positive serum, whereas the gray box represents peptides that reacted only weakly. **(B)** P/N ratio for m1B1–m1B7 in indirect ELISA. **(C)** Schematic diagram of overlapping m2B1–m2B7 short peptides. **(D)** P/N ratio for m2B1–m2B7 in indirect ELISA.

To identify the antigenic regions with good immunogenicity in peptides m1B and m2B, 14 truncated and overlapping short peptides of m1B or m2B were coated onto ELISA plates, and their immunogenicity was probed with PRRSV-positive serum. The peptides m1B1 (562–577), m1B4 (586–601), m1B5 (594–609), and m1B7 (611–627) were recognized by the positive sera (Figures 4A,B). The truncated peptide m2B5 (781–796) reacted well with NADC30-like PRRSV-positive serum, whereas other m2B short peptides displayed only a weak reaction (Figures 4C,D).

### Sequence Conservation Analysis of m1B and m2B

**Figure 5:**
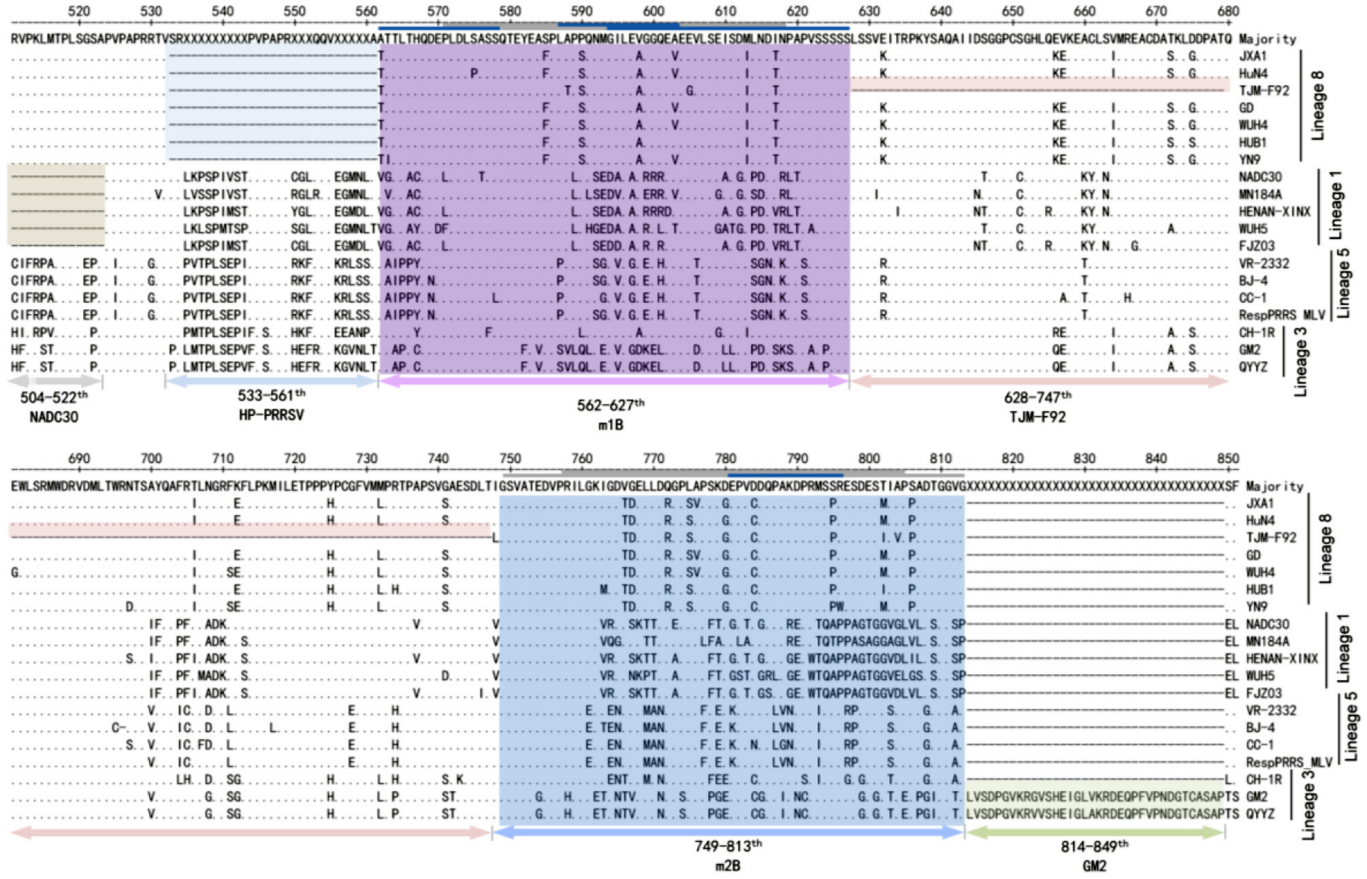
Sequence analysis of m1B and m2B. Representative strains of current circulating PRRSVs or modified live vaccines (MLVs) are shown. The positions of indels in m1B and m2B are labeled below the sequences, whereas overlapping short peptides (m1B1–m1B7 and m2B1–m2B7) are labeled above the sequences. A blue line indicates reactivity between the short peptide and PRRSV-positive serum, whereas a gray line denotes no reactivity.

Sequence alignment of NSP2 revealed that m1B and m2B were conserved within the lineage but diverged from other lineages (Figure 5). The C-termini of m1B1, m1B5, and m1B7 were relatively conserved among lineages, as was part of the middle portion of m2B5; however, in general, the epitope sequences varied considerably among lineages.

### Rescue of PRRSVs Harboring m1B or m2B Deletions

**Figure 6:**
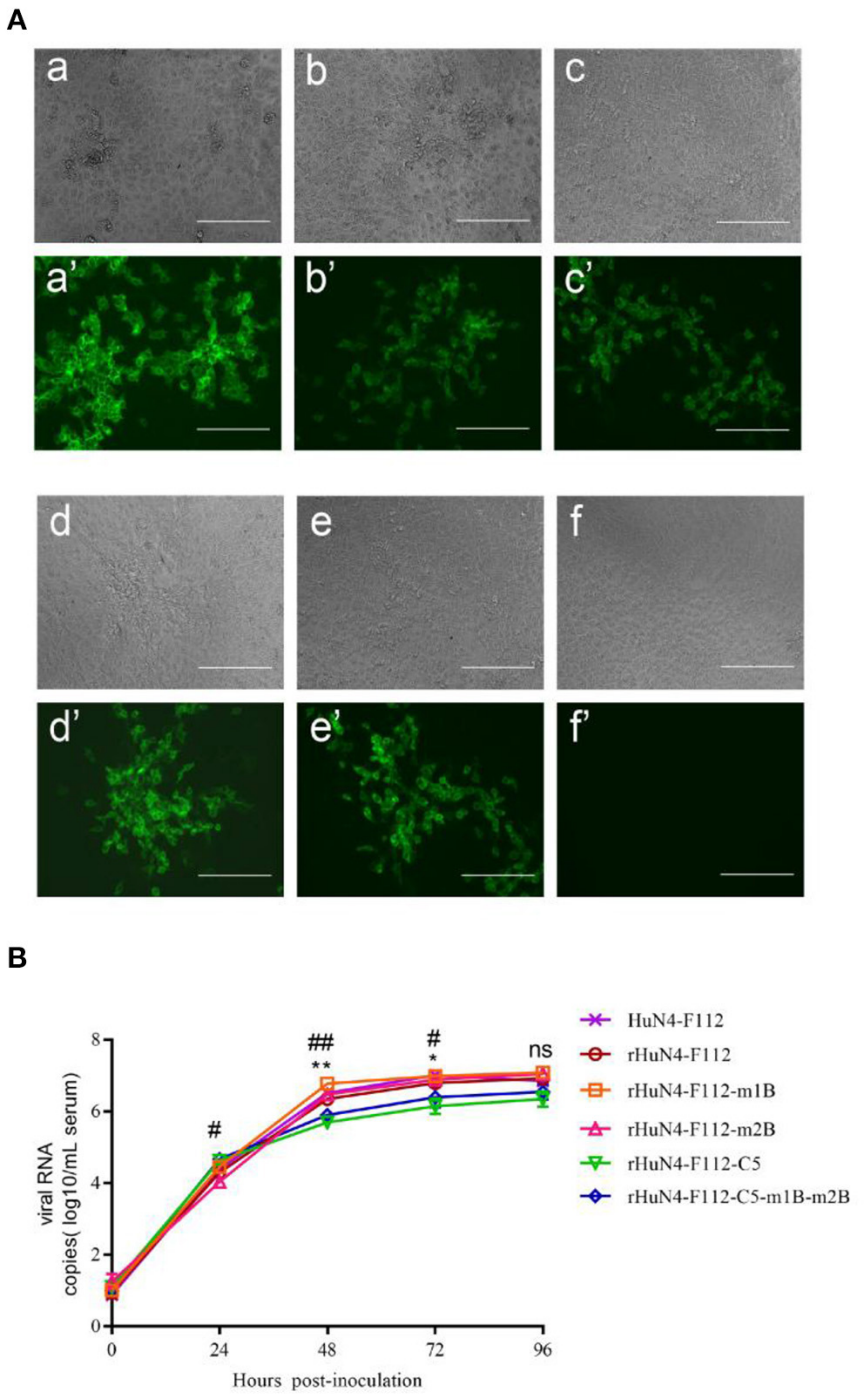
Characterization of PRRSV m1B and m2B deletion mutants. **(A)** CPE and bright fluorescence of deletion-marker strains and parental PRRSV-infected MARC-145 cells. a, rHuN4-F112-m1B; b, rHuN4-F112-m2B; c, rHuN4-F112-C5-m1B-m2B; d, rHuN4-F112; e, rHuN4-F112-C5; f, mock. Bar: 200 μm. **(B)** Growth curves of the parental virus and deletion mutants in Marc-145 cells. Values represent the average of three independent experiments. Error bars indicate standard deviations. Significant differences between the rHuN4-F112-m2B and parental virus are indicated (^#^*P* < 0.05, ^##^*P* < 0.01); significant differences between the rHuN4-F112-m1B and parental virus are indicated (*P < 0.05, **P < 0.01).

Using the infectious HuN4-F112 MLV clone as a template, deletions in the m1B or m2B regions were generated, and the rescue of the corresponding PRRSV deletion mutants (rHuN4-F112-m1B, rHuN4-F112-m2B, and rHuN4-F112-C5-m1B-m2B) was assessed in Marc-145 cells. A cytopathic effect was observed in Marc-145 cells infected with the deletion mutants, as indicated by the bright intracellular immunofluorescence (Figure 6A). All rescued viruses were sequenced at passage 3, and the specific deletions in NSP2 were confirmed.

### Simultaneous Deletion of m1B and m2B Does Not Affect Viral Growth

The titers of rHuN4-F112-C5-m1B-m2B and rHuN4-F112-C5 in Marc-145 cells were 10^5.536^ and 10^5.485^ TCID_50_/ml, respectively. For the single-deletion mutants, the titers of rHuN4-F112-m1B and rHuN4-F112-m2B were 10^7.447^ and 10^7.168^ TCID_50_/ml, respectively, whereas that of their parental rHuN4-F112 virus was 10^7.465^ TCID_50_/ml. The viral growth curve was assessed by RT-qPCR. The copy number of rHuN4-F112-m1B was higher than that of its parental rHuN4-F112 from 24 to 72 h post infection (hpi), but no significant difference at 96 hpi (Figure 6B), similar phenomenon was observed in rHuN4-F112-m2B. The copy number of the double mutant was not significantly different from that of the parental virus rHuN4-F112-C5 at each time point. This result indicates that simultaneous deletion of m1B and m2B at positions 562–627 and 749–813 in NSP2 did not affect the replication of PRRSV in Marc-145 cells.

## Discussion

Porcine reproductive and respiratory syndrome has caused large economic losses in the pig industry in the past and remains a challenging problem in pig farming. Several killed vaccines or MLVs have been licensed and are widely used. Although these vaccines have played an important role in preventing and controlling PRRS epidemics, they are also associated with some limitations, such as limited immunological protection against homologous PRRSVs (28) and difficulty in distinguishing between vaccinated and infected animals (29). We previously described two amino acid deletions in NSP2 of PRRSV during viral passage in Marc-145 cells, which suggested that these or nearby residues can be deleted.

Non-structural protein 2 presents with high antigenicity, and many epitopes have been identified in this protein (30–32), including immunodominant B cell epitopes and some potential T cell epitopes (33). In this study, we identified two immunodominant antigenic regions, m1B and m2B; the results of ELISA indicate that positive serum from different lineages of PRRSV, including HP-PRRSV, C-PRRSV, NADC30-like PRRSV, and NADC34-like PRRSV, could be detected by the m1B + m2B-coated ELISA. Owing to the genetic diversity of PRRSV lineages, the epitope sequences are not relatively conserved among lineages, and this is the reason for the different reaction of the different lineages to these peptides. To avoid possible false negatives caused by antigenicity differences among different strains, the composition of m1B + m2B was recommended for a potential DIVA vaccine. We did not further refine the epitopes, mainly because the reactivity of the refined short peptides was not sufficiently strong, as indicated by their positive-to-negative ratio of ~2. We therefore speculated that several antigenic peptides in m2B are recognized simultaneously by antibodies, and the additive effects could result in a higher reaction signal. Therefore, rather than using individual epitopes, we combined the m1B and m2B peptides when coating the ELISA plates.

Non-structural protein 2 is the largest protein of PRRSV, reaching 1196 residues in VR-2332, the prototype strain of PRRSV-2. Insertions and deletions in NSP2 are frequent (Figure 6), particularly in the middle portion (14), such as in NADC30-like PRRSVs, NADC34-like PRRSVs, and HP-PRRSVs. This finding suggests that NSP2 can tolerate native amino acid indels of exogenous genes. In theory, indels can be repaired via the recombination of two different PRRSV strains; however, no such event has been detected. We analyzed more than 700 PRRSV-2 sequences deposited in GenBank between 1991 and 2020. High-frequency hotspots of inter-lineage recombination were found in NSP9 and the GP2-GP3 region. In contrast, in NSP2, the frequency of inter-lineage recombination is very low in PRRSVs from China or the United States (34). Therefore, the possibility of restoring these mutants to wild-type PRRSV through genetic recombination is very low.

We performed an alignment based on all the available PRRSV NSP2 complete sequences (*n* = 907), and the results revealed extensive natural deletions in the NSP2 of PRRSVs. However, there was no deletion in the m1B or m2B regions of 907 PRRSV strains. Therefore, a differential diagnosis method based on m1B or m2B will reduce false negatives caused by natural deletions. Han et al. (14) identified 324–726 amino acids in the middle region of NSP2 as non-essential for replication and for which a deletion did not affect virus rescue. In another study using the HuN4-F112 infectious clone as a backbone, 25 amino acids were deleted in the non-essential region of NSP2 (at position 508–532), and the dominant epitope of the Newcastle disease virus nucleoprotein (NP49) was inserted in their place to construct a double-marker attenuated vaccine strain (rHuN4-Δ25+NP49) (35). The insertion position, size, and nature of the foreign gene could affect the viability and genetic stability of the obtained virus. Among previous attempts to insert genes encoding the green fluorescent protein (GFP), luciferase, and a Flag tag in the non-essential region of NSP2, only GFP insertion resulted in the rescue of PRRSV particles. With the passage of the virus, the inserted GFP accrued gene mutations and deletions until it lost its activity (36, 37). Thus, the reason why exogenous genes cannot exist in a stable form in NSP2 requires further exploration.

In the present study, two peptides that are universal in NSP2 of PRRSV-2 were found to be immunodominant. This characteristic can be used to monitor antibodies against PRRSV by peptide-coated ELISA. More importantly, PRRSV mutants lacking both peptides multiplied well in Marc-145 cells, suggesting the potential of the two peptides to be used as molecular markers for developing a DIVA vaccine against PRRSV and accompanying differential diagnostic methodology.

## Data Availability Statement

The original contributions presented in the study are included in the article/supplementary material, further inquiries can be directed to the corresponding author/s.

## Author Contributions

T-QA conceived and designed research. D-YL, X-YC, X-XT, Y-BY, and YH conducted experiments. X-HC, Z-JT, and X-YH contributed new reagents or analytical tools. TW and QW analyzed data. D-YL and T-QA wrote the manuscript. All authors read and approved the manuscript.

## Funding

This study was supported by grants from the National Natural Science Foundation of China (32072851 and 31941019) and the State Key Laboratory of Veterinary Biotechnology Research Fund (SKLVBF201902 and SKLVBF202110).

## Conflict of Interest

The authors declare that the research was conducted in the absence of any commercial or financial relationships that could be construed as a potential conflict of interest.

## Publisher's Note

All claims expressed in this article are solely those of the authors and do not necessarily represent those of their affiliated organizations, or those of the publisher, the editors and the reviewers. Any product that may be evaluated in this article, or claim that may be made by its manufacturer, is not guaranteed or endorsed by the publisher.
